# Impaired anti-viral immune response to human rhinovirus 1B infection in chronic allergic airway inflammation does not manifest in asthma exacerbation

**DOI:** 10.1186/1476-9255-10-S1-P20

**Published:** 2013-08-14

**Authors:** Sabine Rochlitzer, Heinz-Gerd Hoymann, Armin Braun, Meike Müller

**Affiliations:** 1 Fraunhofer Institute for Toxicology and Experimental Medicine, Hannover, Germany; 2 Hannover Medical School, Hannover, Germany

## Background

Human rhinovirus is most prominently associated with asthma exacerbations in humans. The U-BIOPRED project of the Innovative Medicine Initiative (IMI) aims to define biomarkers of asthma exacerbations. Within this context, this study aimed to establish a mouse model of asthma exacerbations induced by viral respiratory infection on the background of chronic allergic airway inflammation.

## Materials and methods

BALB/c mice were sensitized intranasally with house dust mite (HDM) extract (25µg in 50µl saline) for five days per week over 7 weeks. HRV1b was inoculated intranasally on the final three challenge days 30 minutes prior HDM application. Additionally, animals were treated with fluticasone proprionate daily prior to HDM challenge in the final week to assess steroid efficacy. 24h after the last combined virus/allergen challenge, pulmonary function (lung resistance, RL) was measured invasively to assess airway hyperresponsiveness (AHR) against aerosolized methacholine (MCh). Bronchoalveolar lavage (BAL) differential cell count and cytokines, lung histology and mediastinal lymph node response were analyzed.

## Results

The HDM sensitized animals developed a marked allergen-induced AHR and eosinophilic airway inflammation compared to the saline control group. Additional rhinovirus infection did not result in an exacerbation phenotype regarding AHR, BAL inflammatory cell counts, draining lymph node cell counts and *ex vivo* proliferative response. However, a satellite control group of naïve mice infected with HRV1B revealed an impaired anti-viral immune response in animals with chronic airway inflammation, indicated by reduced BALF neutrophil cell counts and cytokine levels of IFN-γ, IL-12, IL-1β and TNF-α, as well as *ex vivo* proliferative response of draining lymph node cells.

## Conclusions

The established model of combined chronic airway inflammation and viral infection failed to mimic the symptoms of exacerbating asthma pathology. Nevertheless, this model was able to show *in vivo* the impaired anti-viral immune response due to chronic allergic airway inflammation. The failure to induce exacerbation might be due to inefficient replication of rhinovirus in the mouse, therefore the use of other respiratory viruses might succeed in developing exacerbation models. However, the observed impaired anti-viral response in mice with chronic airway inflammation might reflect the basis for an altered progress of the disease, ultimately resulting in a more severe phenotype.

